# 5-azacytidine affects TET2 and histone transcription and reshapes morphology of human skin fibroblasts

**DOI:** 10.1038/srep37017

**Published:** 2016-11-14

**Authors:** Elena F. M. Manzoni, Georgia Pennarossa, Magda deEguileor, Gianluca Tettamanti, Fulvio Gandolfi, Tiziana A. L. Brevini

**Affiliations:** 1 Laboratory of Biomedical Embryology, Centre for Stem Cell Research; Università degli Studi di Milano, Milan, 20133, Italy; 2 Department of Biotechnology and Life Sciences; Università degli Studi dell’Insubria, Varese, 21100, Italy

## Abstract

Phenotype definition is controlled by epigenetic regulations that allow cells to acquire their differentiated state. The process is reversible and attractive for therapeutic intervention and for the reactivation of hypermethylated pluripotency genes that facilitate transition to a higher plasticity state. We report the results obtained in human fibroblasts exposed to the epigenetic modifier 5-azacytidine (5-aza-CR), which increases adult cell plasticity and facilitates phenotype change. Although many aspects controlling its demethylating action have been widely investigated, the mechanisms underlying 5-aza-CR effects on cell plasticity are still poorly understood. Our experiments confirm decreased global methylation, but also demonstrate an increase of both Formylcytosine (5fC) and 5-Carboxylcytosine (5caC), indicating 5-aza-CR ability to activate a direct and active demethylating effect, possibly mediated via TET2 protein increased transcription. This was accompanied by transient upregulation of pluripotency markers and incremented histone expression, paralleled by changes in histone acetylating enzymes. Furthermore, adult fibroblasts reshaped into undifferentiated progenitor-like phenotype, with a sparse and open chromatin structure. Our findings indicate that 5-aza-CR induced somatic cell transition to a higher plasticity state is activated by multiple regulations that accompany the demethylating effect exerted by the modifier.

DNA methylation is essential for mammalian development, gene regulation, genomic imprinting, and chromatin structure^1^. Changes in methylation allow mature cells of adult organisms to acquire their differentiated state through a gradual loss of potency^2^ and a progressive restriction in their options^3^.

The process is reversible and may be altered by biochemical and biological manipulation, making it an attractive target to reactivate hypermethylated pluripotency genes^4^ and facilitate cell transition to a higher plasticity state^5^.

The epigenetic modifier 5-azacytidine (5-aza-CR) is a chemical analogue of cytosine. It is known to induce reversible cell cycle arrest^6^,^7^ and acts as a direct inhibitor of methyltransferase activity, decreasing methylation in newly synthesised DNA. The molecule substitutes for cytosine, incorporates into DNA and RNA, during replication^8^,^9^, forms covalent adducts with DNA methyltransferase (DNMT) 1, thereby depleting the cells from enzyme activity, causing demethylation of genomic DNA^8^,^10^, as well as gene reactivation^11^. Thanks to its powerful effects, this compound may be used to increase chromatin plasticity and facilitate phenotype changes^12^,^13^. Several studies reported 5-aza-CR ability to facilitate adult somatic cell switch from one phenotype to a different one^14^,^15^,^16^. In particular, we demonstrated that a short exposure to 5-aza-CR allows a transient passage through a plastic chromatin state. This is sufficient to allow a complete directed differentiation of an adult mature cell into a different cell type^17^,^18^,^19^,^20^.

All these studies are very promising because they allow to obtain safe and viral vector free cells that may be used for regenerative medicine. However, the mechanisms underlying 5-aza-CR effects on cell plasticity and differentiation are still poorly understood and need to be better elucidated. In this manuscript we expose 5-aza-CR treated cells either to: 1) embryonic stem cell (ESC) medium (to promote and maintain cell plasticity); 2) pancreatic differentiation (PANCR) medium (to encourage and boost differentiation); 3) standard fibroblast (FB) culture medium (to allow cells to revert to their original phenotype).

We analyze the global methylation changes taking place in cells exposed to the epigenetic modifier and in the different experimental conditions tested. Based on preliminary Whole Transcriptome Analysis, obtained with an Applied Biosystems SOLiD 5500xl Sequencer, we investigate in details changes in transcriptions of the TET family genes, that affect methylation^21^,^22^ and play an essential role in pluripotency regulation of ESC^21^,^22^ and in the very early stage of somatic cell reprogramming toward induced Pluripotent Stem Cells (iPSCs)^23^. We study whether upregulation of TET2 results in increased levels of 5-Formylcytosine (5fC) and 5-Carboxylcytosine (5caC), which are both products of TET2 enzyme-mediated oxidation of 5-methylcytosine. This would suggest the possibility of a direct demethylating mechanism, accompanying the well documented indirect DNMT related action. To further support this hypothesis, we then investigate the effect of siRNA TET2 on the global DNA demethylation caused by 5-aza-CR. We also analyze the expression of histones belonging to the 1, 2 A, 2B, 3 and 4 families. Furthermore, we characterize the ultrastructural phenotypic modifications related to the exposure to 5-aza-CR and to the different culture conditions.

The data presented confirm the well-known effect on methylation but also highlight new cellular targets accompanying 5-aza-CR effects on epigenetic regulation of cell plasticity and differentiation.

## Results

### Global methylation changes in response to 5-aza-CR

**Figure 1:**
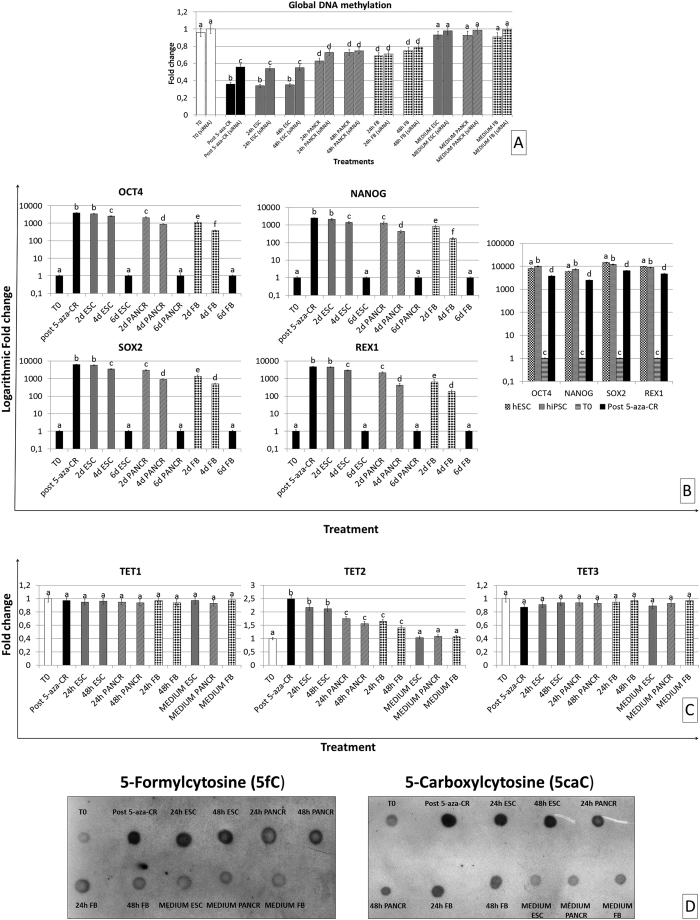
(**a**) Global DNA methylation of cells before and after siRNA transfection, exposed to 5-aza-CR and to different treatments for 24 and 48 hours. Highest expression set to 1 and all other times relative to this. Bars represent the mean ± SD of two independent experiments with three independent replicates, obtained from three different fibroblast primary lines. Different superscripts denote significant differences between groups (P < 0.05). (**b**) 5-aza-CR treatment induced the onset of pluripotency gene expression, increasing the transcription of OCT4, SOX2, NANOG and REX1 (Post 5-aza-CR). Culture in ESC medium for two days (2d ESC) maintained upregulated expression of all the genes, that are turned down by day 6 of culture (6d ESC). 5-aza-CR treated cells (Post 5-aza-CR) display pluripotency gene transcription levels in the range of 45% of those detected in human ESC (hESC) and iPSC (hiPSC). Gene expression levels are reported with the T0 expression set to 1 and all other times relative to this. Different superscripts denote significant differences between groups (P < 0.05). (**c**) Effect of 5-aza-CR on TET gene family transcription. The modifier upregulated TET2, while leaving the other TET members unchanged. Gene expression levels are reported with the T0 expression set to 1 and all other times relative to this. Different superscripts denote significant differences between groups (P < 0.05). (**d**) DNA dot blot analysis of 5-Formylcytosine (5fC) and 5-Carboxylcytosine (5caC) in cells exposed to 5-aza-CR and to different treatments for 24 and 48 hours.

Exposure to 5-aza-CR induced a significant decrease of global DNA methylation (Fig. 1a). In particular, human fibroblasts (T0) treated for 18 hours with the epigenetic modifier (Post 5-aza-CR), showed a significant decrease in DNA methylation. Cells maintained comparable methylation levels when they were cultured in ESC medium for 24 and 48 hours respectively (24 h ESC; 48 h ESC). In contrast, methylation significantly increased in cells exposed to PANCR medium (24 h PANCR; 48 h PANCR) as well as in those returned to FB medium (24 h FB; 48 h FB). No methylation changes were observed in cells that were cultured in the different media, without a previous exposure to 5-aza-CR (MEDIUM ESC; MEDIUM PANCR; MEDIUM FB), indicating that the medium alone is not able to affect DNA methylation levels in differentiated cells.

### Human fibroblasts acquire a transient high plasticity state after epigenetic erasing with 5-aza-CR

Methylation decrease was accompanied by the up-regulation of the ten-eleven translocation 2 (TET2) gene (Fig. 1c, Post 5-aza-CR). However, the modifier did not affect all the TET family members, but rather it exerted a distinct effect on TET2 transcription, while no significant effect was observed for TET1 and TET3.

**Figure 2:**
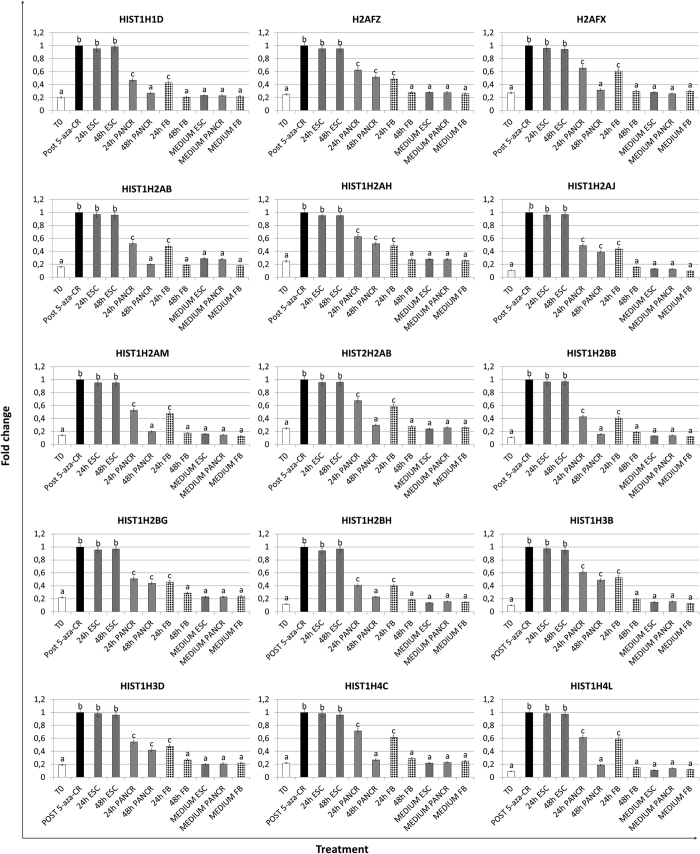
Histone transcription changes in adult human skin fibroblasts exposed to 5-aza-CR and subjected to three different culture conditions for 24 and 48 hours. Transcription was affected by the epigenetic eraser and persisted at high levels in cells cultured in ESC medium (24 h and 48 h ESC). It decreased when post 5-aza-CR cells where exposed to differentiation media. (24 h and 48 h PANCR; 24 h and 48 h FB). Gene expression levels are reported with the highest expression set to 1 and all other times relative to this. Different superscripts denote significant differences between groups (P < 0.05).

5-aza-CR also induced the onset of pluripotency gene expression [POU class 5 homeobox 1 (OCT4), sex determining region Y-box 2 (SOX2), Nanog homeobox (NANOG), and ZFP42 zinc finger protein (REX1)] (Fig. 1b, Post 5-aza-CR). ESC medium maintained high expression levels of all these genes for the first 2 days of culture. By contrast, pluripotency gene expression significantly decreased by day 2, when post 5-aza-CR cells were cultured in differentiation media (Fig. 1b; 2d, 4d, 6d PANCR; 2d, 4d, 6d FB). Furthermore, even when maintained in ESC medium over a longer period of time, cells turned down the transcription of these genes by day 6 of culture (Fig. 1b; 6d ESC).

### TET2 upregulation resulted in 5-Formylcytosine and 5-Carboxylcytosine increase

5-aza-CR induced TET2 upregulation resulted in significant increase of 5fC and 5caC (Fig. 1d and Suppl. Figure 1). These increased levels were also present in cells cultured in ESC medium for 24 h and 48 h (24 h ESC; 48 h ESC), while decreased when cells were transferred to PANCR (24 h PANCR; 48 h PANCR) and FB (24 h FB; 48 h FB) media. No changes were observed in cells that were cultured in the different media, without a previous exposure to 5-aza-CR (MEDIUM ESC; MEDIUM PANCR; MEDIUM FB).

### TET2 downregulation affects 5-aza-CR induced global DNA demethylation

Global DNA demethylation induced by 5-aza-CR was decreased in response to TET2 RNA silencing (Fig. 1a). Significant differences in methylation levels were also detected in cells cultured in ESC medium (24 h ESC; 48 h ESC). No differences were detected in the other groups (T0; 24 h PANCR; 48 h PANCR; 24 h FB; 48 h FB; MEDIUM ESC; MEDIUM PANCR; MEDIUM FB).

### 5-aza-CR modulates expression of histones and histone acetylating enzymes in human fibroblasts

**Figure 3:**
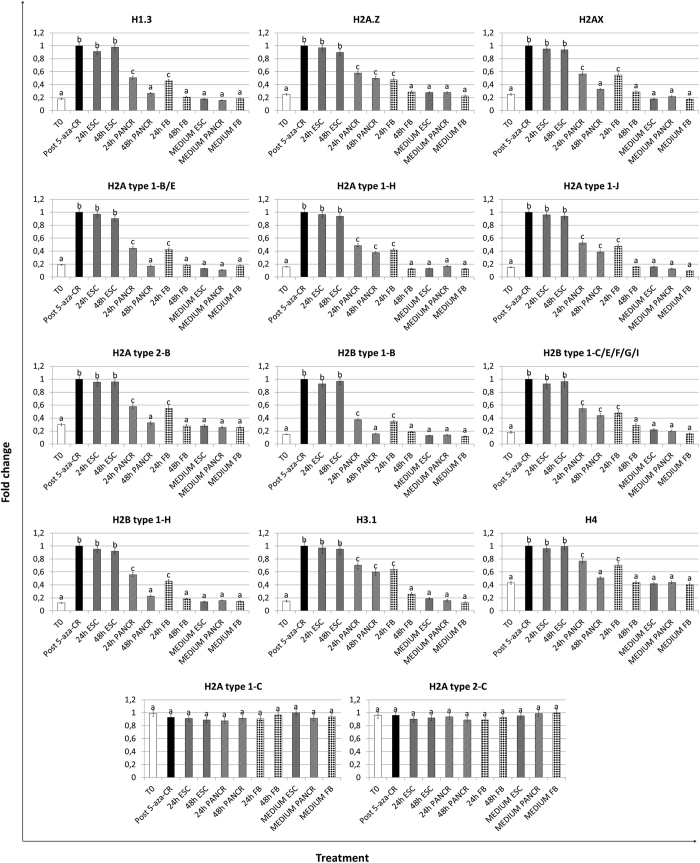
Histone protein levels in adult human skin fibroblasts exposed to 5-aza-CR and subjected to three different culture conditions for 24 and 48 hours. 5-aza-CR significantly increased histone protein concentrations that persisted at high levels in ESC medium (24 h and 48 h ESC). Protein levels decreased when post 5-aza-CR cells were cultured in differentiation media. (24 h and 48 h PANCR; 24 h and 48 h FB). Notably no changes were detected for H2A type 1-C and H2A type 2-C. Different superscripts denote significant differences between groups (P < 0.05).

Expression analysis demonstrated 5-aza-CR ability to significantly upregulate the transcription of histones within the 1, 2, 3 and 4 families, and their encoded protein (Figs 2 and 3). Histones showed a significant increase post 5-aza-CR and persisted at high levels in cells cultured in ESC medium for 24 h and 48 h (24 h ESC; 48 h ESC). In contrast, their expression levels were significantly down-regulated when cells were transferred to PANCR (24 h PANCR; 48 h PANCR) and returned to values comparable to T0 after 48 h in FB medium (48 h FB). No significant change was detected in cells cultured in the different media, without prior exposure to 5-aza-CR.

**Figure 4:**
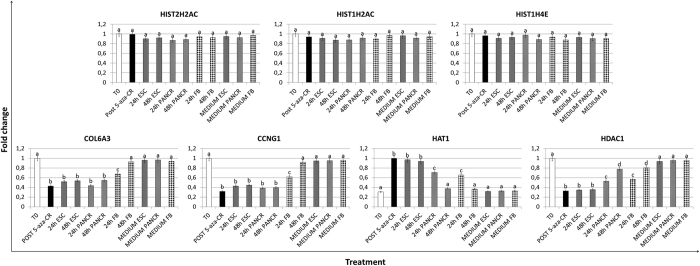
A gene specific effect accompanies 5-aza-CR demethylating action. Exposure to the modifier didn’t affect the transcription of several histones (HIST1H2AC, HIST2H2AC and HIST1H4E), or resulted in downregulation of COL6A3 and CCNG1. Furthermore, a sharp upregulation of HAT1, and a decrease of HDAC1 was detected after exposure to 5-aza-CR. Different superscripts denote significant differences between groups (P < 0.05).

5-aza-CR did not affect the expression of HIST1H2AC (H2A type 1-c) and HIST2H2AC (H2A type 2-C, Figs 3 and 4). Moreover, we detected a parallel downregulation of a fibroblast specific gene (COL6A3) as well as a decreased expression of a transcript involved in cell cycle control (CCNG1). Furthermore, increased expression of histones was supported by the upregulation of the acetylating enzyme Histone acetyltransferase 1 (HAT1) and by the decreased expression of the Histone deacetylase enzyme 1 (HDAC1).

### 5-aza-CR affects cell morphology and chromatin organization

**Figure 5:**
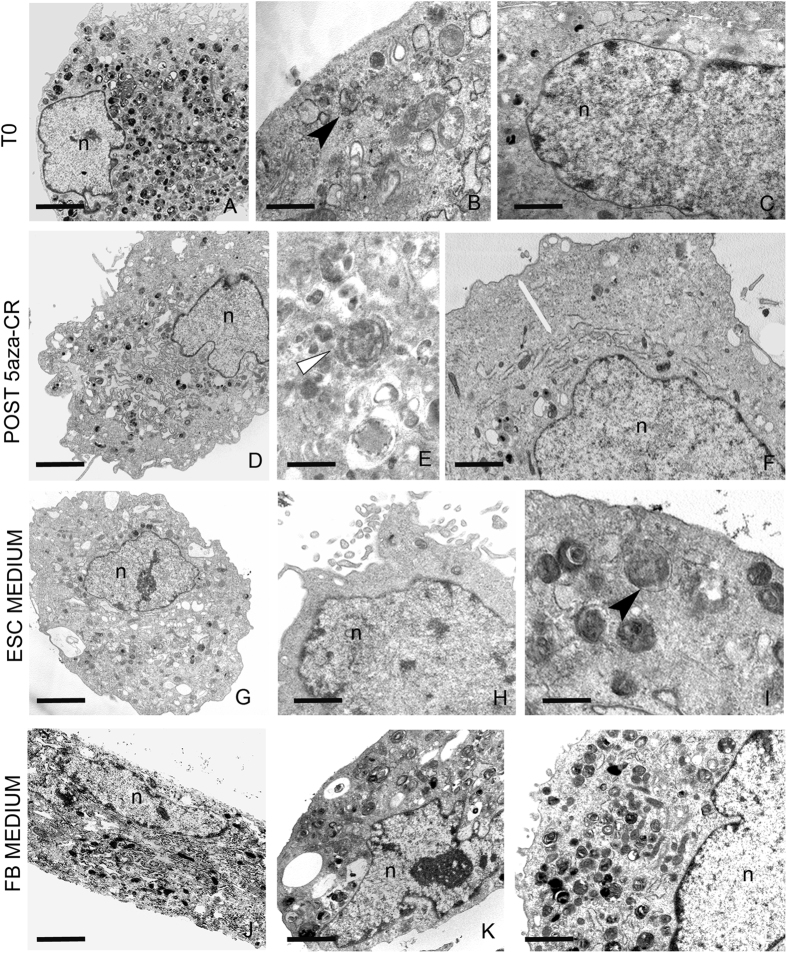
5-aza-CR induced cell plasticity is unravelled by morphological changes. The distinct fibroblastoid morphology (panels a–c) change after exposure to 5-aza-CR (panels d–f) with the acquisition of an undifferentiated progenitor-like phenotype characterized by nuclei (n) showing chromatin decondensation (panels d, f) and autophagic phenomena (panel e, arrowhead). Cells can be characterized by cytoplasmic peripheral ring without organules that are present in the perinuclear area (panel f, white arrow). Post 5-aza-CR cells cultured in ESC medium (panels g–i) maintain the undifferentiated phenotype (n: nucleus; autophagosomes: arrowhead). Post 5-aza-CR cells cultured in FB medium (panels j–l) show re-established features typical of fibroblasts. Scale bars: a, 2.5 μm; b,1 μm; c,1 μm; d, 2 μm; e,0.8 μm; f, 2 μm; g, 4 μm; h, 1.4 μm; i, 0.6 μm; j, 2 μm; k, 2 μm; l, 1.7 μm.

T0 cells displayed a typical fibroblast morphology and were characterized by nuclei with condensed areas close to the nuclear membrane and large vacuoli. The perinuclear compartment contained rough endoplasmic reticulum cisternae (RER), widely scattered mitochondria, vesicles and electron dense granules (Fig. 5 panels a–c).

Count in semi-thin sections demonstrated that 86,31 ± 4,13% cells remarkably changed their morphology after exposure to 5-aza-CR, showing a reduced cell size and either few short microvilli on the plasma membrane or a smoth cell surface. At higher magnification, in the electron-lucent cytoplasm, we could detect large roundish or lobed nuclei that displayed a global chromatin decondensation, mitochondria, RER, empty or filled vacuoles (Fig. 5 panel d), as well as numerous lysosomes and autophagosomes (Fig. 5 panel e). Simultaneously with the autophagic phenomena, a cytoplasmic peripheral ring, less dense and organule-free, became visible (Fig. 5 panel f), which is likely to be responsible for a final reduction in cell size. These morphological features, that are typically related to a high plasticity phenotype^24^, were maintained by 84,24 ± 5,05% and 83,74 ± 5,52% of cells cultured in ESC medium for 24 h and 48 h respectively, as confirmed at higher magnification (Fig. 5 panels g–i), possibly suggesting that, once acquired, the increased plasticity state can be sustained by signals derived from medium.

Interestingly, cells returned to FB medium after exposure to 5-aza-CR re-established the fibroblast phenotype (Fig. 5 panels j–l), indicating that the effect of the modifier is reversible and allows the re-acquisition of the original cell phenotype.

**Figure 6:**
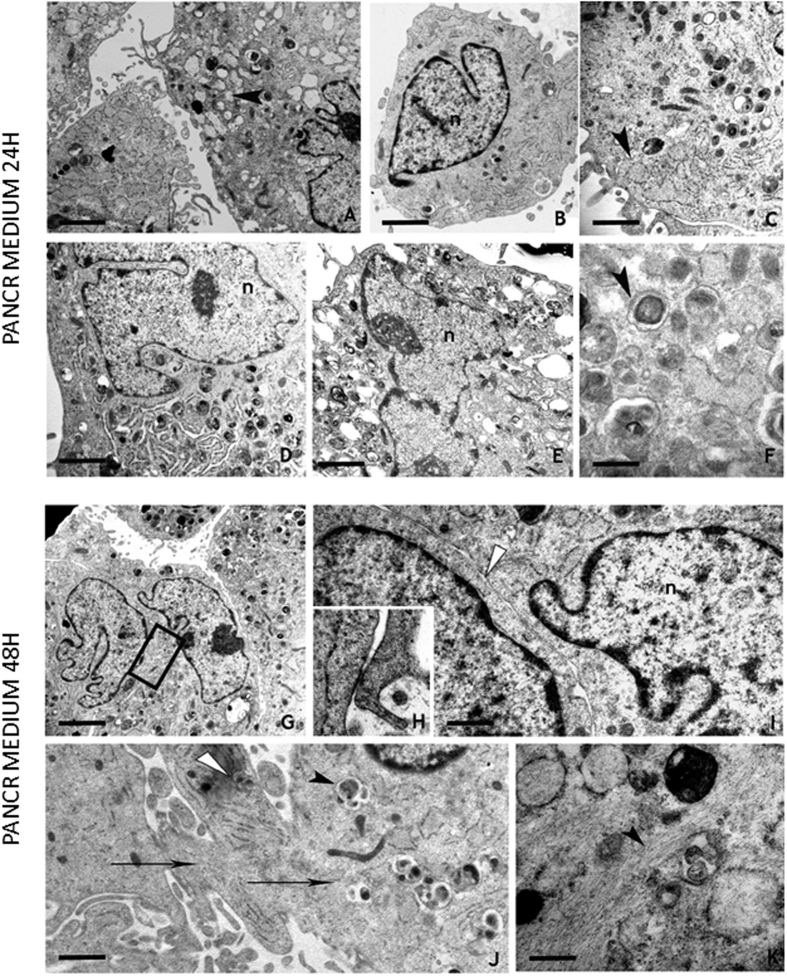
Post 5-aza-CR cells cultured in PANCR medium (panels a–k) show peculiar morphological changes. After 24 hours, cells are characterized by cytoplasmic projections (panel a), indented nuclei (n) (panels a, b, d, e) and large nucleoli. The cytoplasm is occupied by numerous organules, large dilated cisternae of endoplasmic reticulum (panels a, c, arrowheads) and autophagosomes (panel f, arrowhead). After 48 hours, cells can be in close contact (panels g, detail; i, white arrowehead) or tightly joined by gap-like junction (see enlargement in panel h) or can show cytoplasmic bridges (panel j, arrows). In the cytoplasm, phagolysosomes (panel j, arrowhead) and bundles of filaments (panel k, arrowhead) are visible. Scale bars: a, 2 μm; b, 2 μm; c, 3.3 μm; d, 1.7 μm; e, 1.4 μm; f, 0.5 μm; g, 2 μm; i, 1 μm; j, 1.3 μm; k, 0.5 μm.

When post 5-aza-CR cells were transferred to PANCR medium, which has been previously shown to address them towards the pancreatic endoderm differentiation^17^, they adopted a completely different spatial organization. After 24 hours in this medium, 80,12 ± 4,41% cells were variously shaped and showed numerous and long cytoplasmic projections. At higher magnification, nuclei were characterized by indented profiles, clustered dense chromatin close to the nuclear envelope and prominent nucleoli (Fig. 6 panels a, b, d, e). Cytoplasm contained mitochondria, lysosomes, granules and numerous vacuoles which enclosed materials of different electron density (Fig. 6 panels c, d, f). Numerous RER dilated cisternae, wrapping fine reticular network could be appreciated close to the cell surface (Fig. 6 panels a, c). At 48 hours, the same rate of cells had spotted contacts (Fig. 6 panels g, i) or became tightly attached due to the presence of gap-like junctions (Fig. 6 panel h). Cytoplasmic bridges among adjacent cells were visible (Fig. 6 panel j). Vacuoles with heterogeneous material, granules and autophagosomes were present in the inner region of cytoplasm, while bundles of filaments were localized at the periphery of the cells (Fig. 6 panel k).

## Discussion

The epigenetic modifier 5-aza-CR has been shown to incorporate into DNA during cell replication, leading to a global demethylation and gene reactivation^11^. Thanks to this powerful effects, the compound has been used to increase chromatin plasticity and facilitate phenotype changes in different models^12^,^13^,^17^,^18^,^19^,^20^. Although the DNA demethylation capabilities of 5-aza-CR have been well characterized, both *in vitro* and *in vivo*, there is also reason to believe that this drug exhibits alternative mechanisms of action^25^,^26^. Here we confirm the global effect on methylation and, as shown in Fig. 1a, we detect a decreased methylation in fibroblasts exposed to 5-aza-CR. These levels are maintained in cells cultured in ESC medium, suggesting that DNA methylation levels may be modulated by the milieu of the cells, including signals deriving from the medium. These findings are in agreement with Blaschke *et al*.^27^ and Yin *et al*.^28^ that demonstrated changes in DNA methylation in response to specific media formulation and the contained nutritional factors. This possibility is also confirmed by the observation that cells, treated with the modifier, but exposed to FB medium and or PANCR medium, restored their methylation levels. Interestingly, the changes described were accompanied by an increased expression of several genes. Following preliminary NGS analysis of untreated fibroblasts vs. 5-aza-CR exposed cells, we here confirm upregulated transcription of the TET2 gene, which has been recently shown to support 5-aza-CR dependent demethylation process in hepatocarcinoma cell lines^29^. In particular, we show that upregulation of TET2 results in an increase of both 5fC and 5caC (Fig. 1d), indicating 5-aza-CR ability to activate a direct and active demethylating effect, possibly mediated via TET2 protein. Furthermore, the possibility of a synergistic action between direct and indirect demethylation processes is also demonstrated thank to TET2 siRNA experiments, where we demonstrate that downregulation of TET2 protein results in an overall decrease of global DNA demethylation (Fig. 1a), that however still persist in response to 5-aza-CR, possibly due to the unaffected action of 5-aza-CR on DNMTs.

TET2 upregulation is also in agreement with the acquisition of the higher plasticity state detected in post 5-aza-CR cells, since TET2 has been reported to have a role in pluripotency induction and maintenance^30^. Interestingly, 5-aza-CR did not affect all the members of the TET family, but rather it exerted a distinct effect on TET2, leaving the other members unchanged. Based on previous observations, showing that TET2 does not affect methylation levels at gene promoters and transcription start sites, but, in contrast, mainly regulates gene bodies and exon boundaries, our results suggest the possibility of a specific effect of the modifier on the latter.

Exposure to 5-aza-CR also caused the onset of pluripotency gene expression, such as OCT4, NANOG, REX1 and SOX2 (Fig. 1b). As shown in Fig. 1b, these genes were induced by 5-aza-CR, displaying transcription levels in the range of 45% of those detected in human ESCs and iPSCs. This result is also consistent with the molecule ability to reactivate previously silent genes and to alter the differentiation state of eukaryotic cells, as well as with recent data showing that 5-aza-CR demethylation of one or more loci facilitates a rapid and stable transition of partially reprogrammed iPSCs to a fully reprogrammed state^31^.

Interestingly, culture in ESC medium maintained upregulated expression of all the genes described, suggesting the acquisition of a high plasticity state. However, this condition was transient and reversible, since expression of pluripotency related genes decreased gradually when post 5-aza-CR cells where exposed to differentiation media (24 h and 48 h PANCR; 24 h and 48 h FB). Furthermore, even when maintained in ESC medium over a longer period of time, cells turned down the transcription of these genes by day 6 of culture (Fig. 1b).

The expression of histones belonging to 1, 2, 3 and 4 families was also affected by the exposure to the epigenetic eraser. The genes and their encoded proteins showed increases (between 3 to 11 fold), in cells treated with 5-aza-CR, and persisted at high levels in cells cultured in ESC medium for 24 and 48 hours (Figs 2 and 3). The effect on transcription was evident in replication-dependent as well as in replication-independent histones, indicating that it may not only be a secondary indirect consequence of 5-aza-CR on the cell cycle. Furthermore, histones belonging to the same cluster was either upregulated or unchanged by 5-aza-CR, suggesting a histone specific effect rather than a cluster specific one. We have no ready explanation for this, although it is known that separate sets of histone genes are transcribed in a precise temporal sequence during development and differentiation of lower species^32^. On the other hand, the contribution of histone transcription to the maintenance of high plasticity versus differentiation in mammalian is presently still to be fully understood and further studies are required.

We postulate that the 5-aza-CR effect on histone gene transcription is unlikely to be accounted for a general demethylating effect, since exposure to the modifier, resulted in parallel downregulation of fibroblast specific genes (COL6A3) as well as of transcripts involved in the control of cell cycle (CCNG1) and did not affect the expression of several histones genes (HIST1H2AC, HIST2H2AC and HIST1H4E, Fig. 4). In agreement with this hypothesis, Komashko *et al*. demonstrated that several changes in gene expression after 5-aza-CR treatment were related to an alternative DNA-hypomethylating independent effect^33^. Furthermore, consistent with the idea that this epigenetic modifier has additional modes of action, several works indicated that 5-aza-CR can induce expression of genes lacking CpG methylation^34^,^35^. Interestingly, Takebayashi *et al*. demonstrated a DNA methylation independent mechanism, directly related to an increase in histone hyperacetylation^25^. This is also in agreement with our observation that HAT1 transcription was significantly higher in cells exposed to 5-aza-CR, suggesting the possible correlation among the exposure to the modifier, the overexpression of HAT1, the consequent histone hyperacetylation and increased transcription. This possibility is further supported by the parallel decreased expression of HDAC1 (Fig. 4), reported in our manuscript. It is also coherent with previous works that show how modulation of histone acetylation is an integral component of gene expression control^36^ and demonstrate a key role of the specific enzymatic activity of HDACs in transcriptional regulation^37^.

Morphological analysis further unravelled the impact of 5-aza-CR on cell plasticity and differentiation. In particular, it is interesting to underline that adult skin fibroblasts changed after 5-aza-CR exposure, became smaller in size and reshaped into an undifferentiated progenitor-like phenotype. In this condition, cells showed a nuclei/cytoplasm ratio similar to what previously described in hESCs^38^ and in hiPSCs obtained from human dermal fibroblasts^39^. 5-aza-CR increased plasticity was also accompanied by autophagic phenomena, that have been reported to play a critical role in the maintenance of adult stem cells^40^, possibly reducing senescence via the active removal of oxigen reactive species^41^. Interestingly the presence of autophagic vacuoles have been previously reported in ultrastructural analysis of hiPSC^42^. Furthermore, post 5-aza-CR cell nuclei were characterized by sparse chromatin and appeared devoid of compact formations. This is consistent with the observation that an open chromatin structure is required for a high plasticity state^43^ and is a common feature of iPSC and, more in general, one of the hallmark of stem cells^43^,^44^. Interestingly, this condition was maintained by ESC medium, possibly suggesting that, once acquired, the decreased methylation state can be sustained by factors contained in the medium such as LIF, which has been recently demonstrated to induce murine cell hypomethylation^45^. The acquisition of a transient high plasticity state is also consistent with post 5-aza-CR cell ability to respond to differentiation protocols and acquire a differentiated phenotype^17^,^18^,^19^,^20^. Indeed, when 5-aza-CR treated cells were transferred to PANCR medium, which address them towards the pancreatic endoderm differentiation^17^, they adopted a different phenotype. Nuclei were characterized by indented profiles, prominent nucleoli and clustered dense chromatin, mostly locating close to the nuclear envelope. These morphological changes, involving a packed chromatin conformation, and its clustering near the nuclear envelope, have been previously described and considered to be distinctive of a differentiated state^24^ and suggest that post 5-aza-CR cells may reverse their high plasticity state and respond to differentiation stimuli. This is consistent with the methylation and transcriptional changes described above and indicates that the mechanisms exerted by the modifier have also deep effects at the phenotype level. Such hypothesis is further supported by the observation that the majority of cells showed gap-like junctions and cytoplasmic bridges, all features required to guarantee a correct differentiation processes^46^,^47^.

The reversibility of the effects exerted by 5-aza-CR, was confirmed by the re-acquisition of the original phenotype when cells were returned to FB medium, indicating that a short exposure to the modifier allows a transient high plasticity chromatin state, sufficient to allow cell reshaping.

Altogether, the data presented in this manuscript, indicate 5-aza-CR ability to increase TET2 gene expression, modulate transcription of histones and histone acetylating enzymes, which result in specific morphological changes. Moreover, the results obtained, also suggest the molecule ability to interfere with DNA methylation through a direct TET2-mediated mechanism, that accompanies the well-known indirect DNMT related one.

## Experimental Procedures

All chemicals were purchased from Life Technologies (Italy) unless otherwise indicated.

### Ethics statement

Adult human skin fibroblast primary lines were kindly donated by Dr. Gianpaolo Zerbini (Scientific Institute San Raffaele). Cells were isolated from adult patients aged between 35 and 49 years, after written informed consent and approved by the Ethical Committee of the Department of Internal Medicine of the University of Genoa. All the methods in our study were carried out in accordance with the approved guidelines.

### Skin fibroblast culture (FB)

Three adult skin fibroblast primary lines were isolated from human healthy patients and grown in standard culture medium (FB), consisting of DMEM with 20% (vol/vol) Fetal Bovine Serum (FBS), 2 mM glutamine (Sigma), and antibiotics (Sigma). Cells were passaged twice a week in a 1:3 ratio. All experiments were performed in triplicate.

### 5-aza-CR treatment

Fibroblasts were plated in 4-well multidish (Nunc) previously treated with 0.1% gelatin (Sigma) at concentration of 7.8 × 10^4^ cell/cm^2^. They were then incubated with 1 μM 5-aza-CR (Sigma) for 18 hours. Concentration and time of exposure were selected according to our previous work^17^.

### Experimental design

At the end of 5-aza-CR treatment cells were divided in three experimental groups and cultured for 24 and 48 hours, 4 and 6 days. In the first group, cells were returned in FB medium. The second group of cells were cultured in medium specific for pluripotency maintenance (ESC). Cells of the last group we differentiated toward pancreatic lineage (PANCR).

### Medium for pluripotency maintenance (ESC)

Cells were cultured in DMEM low glucose/ Ham’s F-10 Nutrient Mix (1:1) supplemented with 10% KnockOut Serum Replacement, 5% FBS, 1% antibiotics (Sigma), 0.1 mM β- mercaptoetanol (Sigma), 2 mM glutamine (Sigma), 1 mM MEM Non-Essential Amino Acids, 1% Nucleoside mix, 20 ng/ml Recombinant Human FGF basic (bFGF) and 10^3^ units/ml ESGRO (LIF)^48^.

### Medium for pancreatic induction (PANCR)

Cells were cultured in Dulbecco’s Modified Eagle Medium: Nutrient Mixture F-12 (DMEM/F12) with 1% B27, 1% N2, 0.1 mM β- mercaptoetanol (Sigma), 2 mM glutamine (Sigma), 1 mM MEM Non-Essential Amino Acids, 0.05% bovine serum albumin (BSA, Sigma), and with 30 ng/mL activin A.

### siRNA transfection

The targeting siRNA used in the present study was TET2 siRNA (Santa Cruz) and siRNA induced protein knock-down was carried out as previously described^49^. More in detail, cells were transfected 24 hours before exposure to the modifier and during 5-aza-CR treatment, with 90 pM of siRNA, using Lipofectamine RNAi MAX Reagents diluted in Opti-MEM® Medium, following the manufacturer’s instructions. For negative controls, cells were incubated with Lipofectamine alone or with Lipofectamine plus 90 pM non-silencing siRNAs. The success of the silencing occurrence was assessed by monitoring the decrease of TET2 protein. After transfection global DNA methylation was assessed as described below. All experiments were performed three times in triplicate samples.

### Global methylation analysis

Genomic DNA was extracted from untreated fibroblasts and after their siRNA transfection at different time points: T0, after 5-aza-CR treatment (Post 5-aza-CR), 24 and 48 hours after culture in fibroblast (FB), ESC or pancreatic induction (PANCR) media. PureLink® Genomic DNA Kits was used according to the manufacturer’s instructions. DNA was converted to single-stranded DNA by incubation at 95 °C for 5 min, followed by rapid chilling on ice. Samples were then digested to nucleosides by incubating the denatured DNA with nuclease P1 for 2 h at 37 °C in 20 mM sodium acetate (pH 5.2). Alkaline phosphatase was added and incubated for 1 h at 37 °C in 100 mM Tris (pH 7.5). After centrifugation, the supernatant was used for ELISA assay using Global DNA Methylation ELISA Kit (5′-methyl-2′-deoxycytidine Quantitation; CELL BIOLABS) according to the manufacturer’s protocol.

### DNA dot blot

Genomic DNA was extracted with PureLink® Genomic DNA Kits as previously described. DNA concentration was assessed with NanoDrop 8000 (Thermoscientific). Aliquots of 200 ng total DNA were prepared in a total volume of 2 μL per sample and spotted onto nylon membranes (Hybond-N+, Amersham). The DNA spots were air dried for 15 min, UV-crosslinked for 1 min and probed with primary antibodies against 5-Carboxylcytosine (Active Motif, 1:1500) and 5-Formylcytosine (Active Motif, 1:1500). Dots were visualized with a WesternBreeze chemiluminescent kit. Signal intensity was quantified by densitometric analysis, using the Image J analysis software (National Institutes of Health).

### Gene expression analysis

RNA was extracted with the TaqManGene Expression Cells to Ct kit (Applied Biosystems), and DNase I was added in lysis solution at 1:100 concentration, as indicated by the manufacturer’s instructions. Predesigned gene-specific primer and probe sets from TaqManGene Expression Assays (Applied Biosystems) were used for gene study (Table 1). PCR runs and fluorescence detection were carried out in a 7500 Real-Time PCR System (Applied Biosystems). β-actin was used as internal standard. For each individual gene, the number of amplification cycles for the fluorescent reporter signal to reach a common threshold value (Ct) were estimated and then normalized against the Ct value obtained for β-actin of the same sample to give the ΔCt value.

### Quantification of histone protein levels

Cells were detached from the culture dish with trypsin and collected by centrifugation. They were then washed three times with cold PBS (1x) and subjected to ultrasonication. After centrifugation and dilution, samples were used for ELISA assay using the commercially available Enzyme-linked Immunoassorbent Assay Kit (MyBioSource) for the specific histone (Table 2), according to the manufacturer’s protocol. Optical density (O.D.) measurements were done using a Multiskan™ FC Microplate Photometer (Thermo Fisher) at the wavelength of 450 nm. Protein concentration in the samples was determined by comparing the O.D. of the samples to the standard curve.

### Morphological analysis

Cells were analyzed at different time points: immediately before exposure to 5-aza-CR (T0), after 5-aza-CR treatment (Post 5-aza-CR), 24 and 48 hours after culture in standard fibroblast (FB), in ESC or in pancreatic induction (PANCR) media.

### Electron microscopy

Samples were fixed for 2 hours in 0.1 M cacodylate buffer pH 7.2, containing 2% glutaraldehyde. Specimens were then washed in the same buffer and post-fixed for 2 hours with 1% osmic acid in cacodylate buffer. After standard serial ethanol dehydration, specimens were embedded in an Epon-Araldite 812 mixture. Sections were obtained with a Reichert Ultracut S ultratome (Leica, Austria). Semi-thin sections were stained by conventional methods (crystal violet and basic fuchsin) and observed under a light microscope (Olympus, Japan) to score cells for different morphological changes. Thin sections were stained by uranyl acetate and lead citrate and observed with a Jeol 1010 EX electron microscope (Jeol, Japan).

### Statistical analysis

Statistical analysis was performed using Student t-test (SPSS 19.1; IBM). Data were presented as mean ± standard deviation (SD). Differences of p ≤ 0.05 were considered significant and were indicated with different superscripts.

## Additional Information

**How to cite this article**: Manzoni, E. F.M. *et al*. 5-azacytidine affects TET2 and histone transcription and reshapes morphology of human skin fibroblasts. *Sci. Rep*. **6**, 37017; doi: 10.1038/srep37017 (2016).

**Publisher’s note:** Springer Nature remains neutral with regard to jurisdictional claims in published maps and institutional affiliations.

## Supplementary Material

Supplementary Information

## Figures and Tables

**Table 1:** List of primers used for quantitative PCR analysis of human cells.

GENE	DESCRIPTION	CATALOG NO.
ACTB	Actin, beta	Hs01060665_g1
CCNG1	Cyclin G1	Hs00171112_m1
COL6A3	collagen, type VI, alpha 3	Hs00915125_m1
H2AFX	H2A histone family, member X	Hs00266783_s1
H2AFZ	H2A histone family, member Z	Hs01888362_g1
HAT1	histone acetyltransferase 1	Hs00186320_m1
HDAC1	histone deacetylase 1	Hs02621185_s1
HIST1H1D	histone cluster 1, H1d	Hs00271187_s1
HIST1H2AB	histone cluster 1, H2ab	Hs01001083_s1
HIST1H2AC	histone cluster 1, H2ac	Hs00374312_s1
HIST1H2AH	histone cluster 1, H2ah	Hs00544732_s1
HIST1H2AJ	histone cluster 1, H2aj	Hs04191486_s1
HIST1H2AM	histone cluster 1, H2am	Hs01920904_s1
HIST1H2BB	histone cluster 1, H2bb	Hs00606684_s1
HIST1H2BG	histone cluster 1, H2bg	Hs00374317_s1
HIST1H2BH	histone cluster 1, H2bh	Hs00374322_s1
HIST1H3B	histone cluster 1, H3b	Hs00605810_s1
HIST1H3D	histone cluster 1, H3d	Hs00371415_s1
HIST1H4C	histone cluster 1, H4c	Hs00543883_s1
HIST1H4E	histone cluster 1, H4e	Hs00374346_s1
HIST1H4L	histone cluster 1, H4l	Hs00361930_s1
HIST2H2AB	histone cluster 2, H2ab	Hs00602439_s1
HIST2H2AC	histone cluster 2, H2ac	Hs00543838_s1
NANOG	Nanog homeobox	Hs02387400_g1
OCT4	POU class 5 homeobox 1	Hs00999632_g1
REX1	ZFP42 zinc finger protein	Hs00399279_m1
SOX2	Sex determining region Y-box 2	Hs01053049_s1
TET1	tet methylcytosine dioxygenase 1	Hs00286756_m1
TET2	tet methylcytosine dioxygenase 2	Hs00325999_m1
TET3	tet methylcytosine dioxygenase 3	Hs00379125_m1

**Table 2:** List of ELISA kits used for quantification of histone protein levels.

PROTEIN	DESCRIPTION	COMPANY	CATALOG NO.
H2AX	H2A histone family, member X(H2AFX), ELISA Kit	MyBioSource	MBS9335115
H2A.Z	H2A histone family, member Z(H2AFZ), ELISA Kit	MyBioSource	MBS2025145
H1.3	Histone cluster 1, H1d (HIST1H1D), ELISA Kit	MyBioSource	MBS914357
H2A type 1-B/E	Histone Cluster 1, H2ab (HIST1H2AB), ELISA Kit	MyBioSource	MBS9321468
H2A type 1-C	Histone cluster 1, H2ac (HIST1H2AC), ELISA Kit	MyBioSource	MBS9333153
H2A type 1-H	Histone cluster 1, H2ah (HIST1H2AH), ELISA Kit	MyBioSource	MBS9320153
H2A type 1-J	Histone cluster 1, H2aj (HIST1H2AJ), ELISA Kit	MyBioSource	MBS9343145
H2A type 1	Not commercially available		
H2B type 1-B	Histone cluster 1, H2bb (HIST1H2BB), ELISA Kit	MyBioSource	MBS9317197
H2B type 1-C/E/F/G/I	Histone cluster 1, H2bc (HIST1H2BG), ELISA Kit	MyBioSource	MBS9324014
H2B type 1-H	Histone cluster 1, H2bh (HIST1H2BH), ELISA Kit	MyBioSource	MBS9319723
H3.1	Histone cluster H3.1 (HIST1H3A), ELISA Kit	MyBioSource	MBS937951
H4	Histone H4 (HIST1H4A), ELISA Kit	MyBioSource	MBS2884038
H2A type 2-B	Histone cluster 2, H2ab (HIST2H2AB), ELISA Kit	MyBioSource	MBS9338734
H2A type 2-C	histone cluster 2, H2ac (HIST2H2AC), ELISA Kit	MyBioSource	MBS900872
