# Supplementary material for: 5-azacytidine affects TET2 and histone transcription and reshapes morphology of human skin fibroblasts
Source: Sci Rep. 2016 Nov 14;6:37017. doi: 10.1038/srep37017 (PMC5107985; doi:10.1038/srep37017)
Supplement: Supplementary Information [file srep37017-s1.pdf]

Supplementary information for:

5-azacytidine affects TET2 and histone transcription and reshapes morphology of human skin fibroblasts

Elena F.M. Manzoni, Georgia Pennarossa, deEguileor Magda, Gianluca Tettamanti, Fulvio Gandolfi and Tiziana A.L. Brevini

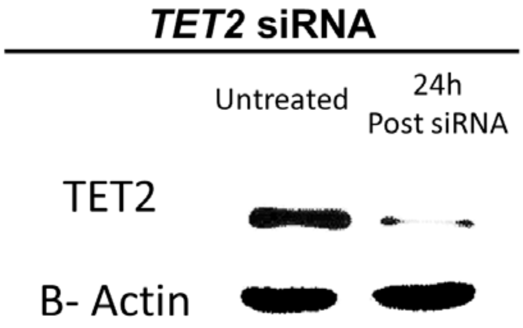

Supplementary Figure S1: TET2 silencing was demonstrated by the decrease of protein level after siRNA treatment.

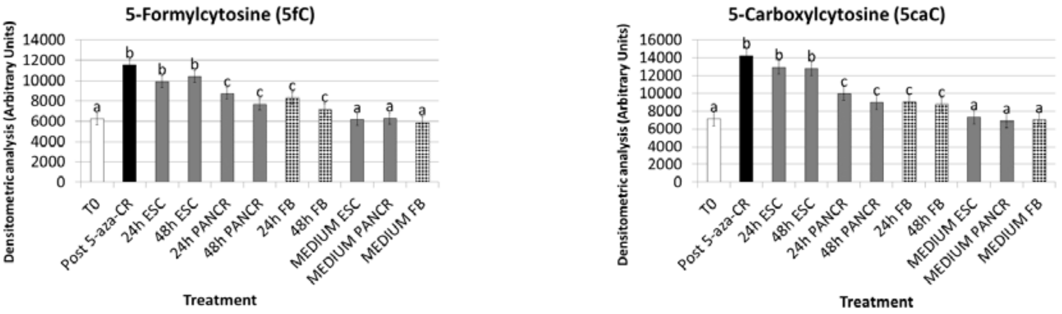

Supplementary Figure S2: DNA dot blot analysis of 5-Formylcytosine (5fC) and 5-Carboxylcytosine (5caC) in cells exposed to 5-aza-CR and to different treatments for 24 and 48 hours. Histogram represents dot-blot signal intensity quantified by densitometric analysis. Bars represents the mean ± SD of three independent replicates. Different superscripts denote significant differences between groups (P<0.05).

Supplementary Table S1. Counts per million (CPM) mapped read values for TET2 and histones in untreated fibroblasts (T0) and 5-aza-CR treated cells (Post 5-aza-CR) obtained through NGS analysis. Quality filtered reads were mapped against the human genome GRCh37 / hg19 using the Lifetech Lifescope ver. 2.5.1 software in paired-end mode. An expression value, represented by the number of mapped sequences (gene counts), was assigned to each target RefSeq gene by the analysis pipeline.

**GENE**	**T0 CPM**	**Post 5-aza-CR CPM**
H2AFX	35.8	141.14
H2AFZ	14.87	60.82
HIST1H1D	18.52	122.70
HIST1H2AB	5.46	29.73
HIST1H2AH	4.67	37.41
HIST1H2AJ	2.31	23.30
HIST1H2AM	4.25	26.10
HIST1H2BB	14.05	43.10
HIST1H2BG	24.30	94.73
HIST1H2BH	2.95	20.15
HIST1H3B	9.68	108.98
HIST1H3D	21.88	99.57
HIST1H4C	22.86	101.56
HIST1H4L	6.79	50.58
HIST2H2AB	4.67	27.50
TET2	17.73	39.49
